# Supplementary material for: Worldwide Traceability of Antibiotic Residues from Livestock in Wastewater and Soil: A Systematic Review
Source: Animals (Basel). 2021 Dec 28;12(1):60. doi: 10.3390/ani12010060 (PMC8749557; doi:10.3390/ani12010060)
Supplement: Supplementary file 1 [file animals-12-00060-s001.zip › animals-1472926-supplementary.pdf]

animals MDPI

Supplementary material

Table S1. Number of studies reporting use of antibiotics per family and active ingredient, by livestock production, soil, and wastewater

Antibiotic class and active ingredients	Total studies (N)	Livestock	Soil	Wastewater
**Penicillin**				
Natural Penicillins	15	3, 4, 5, 7, 8, 13, 14, 15, 54, 55, 68, 106, 120, 133, 152		
Aminopenicillins	9	2,6, 10, 11, 12, 15, 40, 151	1	
Antistaphylococcal Penicillins	4	15, 40, 55		48
**Tetracyclines**				
Chlortetracycline	10	20, 22, 15, 36, 107	17, 72, 83	31, 28
Doxycycline	5	15, 55, 107		31, 28
Oxytetracycline	30	8, 21, 10, 12, 27, 32, 15, 34, 36, 40, 55, 61, 68, 78, 107, 112, 118, 120, 149, 150	16, 18	28, 29, 31, 38, 132
Tetracycline	45	19, 3, 23, 26, 33, 35, 36, 41, 54, 55, 70, 74, 75, 76, 79,101, 107, 108, 121, 122	80, 81, 82, 84, 86, 109, 110, 131, 154, 155, 156	30, 31, 37, 28, 101, 103, 146, 147, 148, 153
**Aminoglycosides**				
Aminoglycosides	14	3, 4, 2, 5, 38, 39,15, 37, 54, 55, 77, 143, 144, 145		
**Macrolides**	1			104
Macrolides C14	3	15,		37, 48
Macrolides C16	7	40, 9, 41, 15, 55		31, 48
**Sulfonamides**	65	25, 17, 2, 42, 43, 21, 23, 24, 12, 50, 51, 52, 39, 15, 35, 53, 54, 55, 64, 65, 79, 87, 88, 91, 92, 93, 94, 95, 97, 98, 99, 100, 157, 158 123, 124, 125, 126, 127, 128, 129, 130	72, 73, 80, 81, 82, 84, 89, 90, 96, 110, 111, 136, 142	31, 44, 45, 46, 47, 48, 49, 37, 67, 103
Diaminopyrimidines	1			31
**Quinolones**	11	27, 35, 66, 79, 113	82, 85, 102, 164, 165	59
Quinolones 1G	5	56, 27, 15	81, 135	
Quinolones 2G (Fluoroquinolones)	24	24, 25, 54, 55, 15, 37, 28, 71, 114, 115, 116, 117, 119, 120, 134, 138, 139, 140, 159, 160		48, 57, 58, 141
**Polypeptides**	2	41, 15		
Polypeptides cyclic	1	15		
**Cephalosporins**				
Cephalosporin 3G	3	40, 55, 15		

Cephalosporin 4G	1	15		
Phenicols	14	16, 43, 12, 15, 62, 63, 69, 99, 161	105, 162, 163	37, 60
Lincosamides	7	25,15, 25, 9, 137	16	31
Pleuromutilins	1	21		
Ionophores	1	15		
Streptogramins	1	15		

References listed in supplemental Table S1

1. Subbiah, M.; Mitchell, S.M.; Ullman, J.L.; Call, D.R. β-Lactams and Florfenicol Antibiotics Remain Bioactive in Soils While Ciprofloxacin, Neomycin, and Tetracycline Are Neutralized. *Appl. Environ. Microbiol.* **2011**, *77*, 7255–7260.
2. Salas, Z.P.; Calle, E.S.; Falcón, T.N.; Pinto, J.C.; Espinoza, B.J. Determination of Betalactamic Antibiotic Residues Using an Immunoenzymatic Assay in Milk of Cows Treated against Mastitis. *Rev. De Investig. Vet. Del Perú (RIVEP)* **2013**, *24*, 252–255.
3. Velázquez, F.; Pérez, M.; González, R. Investigación de Residuos de Antibióticos En Leche Pasteurizada y Envasada Que Se Consume En El Área Metropolitana. *Salud Pública De México* **1980**, *22*, 91–99.
4. Pozo-Lora, R.; Herrera-Marteache, A.; Polo-Villar, L.M.; Lopez-Gimenez, R.; Jodral-Villarejo, M.; Iglesias-Perez, J. Investigations of Antibiotic Residues Present in Fluid Milk in the Southern Region of Spain. *Arch. Zootec.* **1977**, *26*,125–145.
5. Cruz-Alamilla, M.; Pérez-Domínguez, M.; Velázquez, F. Frecuencia de La Contaminación de La Leche Disponible En El Valle de México Con Estreptomicina, Tetraciclina y Penicilina. *Salud Pública De México* **1986**, *28*, 438–442.
6. Chowdhury, S.; Hassan, M.M.; Alam, M.; Sattar, S.; Bari, M.S.; Saifuddin, A.K.M.; Hoque, M.A. Antibiotic Residues in Milk and Eggs of Commercial and Local Farms at Chittagong, Bangladesh. *Vet. World* **2015**, *8*, 467.
7. Rathe, H.A.A.; Al-Shaha, O.M. Detection of Antibiotic Residues in Milk and Milk Products of Cattle in Dairy Farms in Baghdad Region. *J. Entomol. Zool. Stud.* **2017**, *5*, 1797–1802.
8. Kaya, S.E.; Filazi, A. Determination of Antibiotic Residues in Milk Samples. *Kafkas Univ Vet Fak Derg* **2010**, *16* (Suppl. A), S31–S35.
9. Faridi, H.; Ahmadi, E. Rheological Evaluation of Chicken Meat Parts under Various Antibiotic Treatments Prior and Post Cooking Process. *J. Food Meas. Charact.* **2015**, *9*, 195–205.
10. Al-Gendy, H.A.; Hasanen, F.S.; Salem, A.M.; Nada, S.M. Assessment of Oxytetracycline and Ampicillin Residues in Sheep Carcasses. *Banha. Vet. Med. J.* **2014**, *27*, 188–196.
11. Hekster, Y.A.; Baars, A.M.; Vree, T.B.; Van Klingeren, B.; Rutgers, A. Comparison of the Determination of β-Lactam Antibiotic Drugs in Plasma of Rabbits by Means of HPLC and of a Microbiological Assay. *Pharm. Weekbl.* **1979**, *1*, 695–700.
12. Schmidt, A.S.; Bruun, M.S.; Dalsgaard, I.; Pedersen, K.; Larsen, J.L. Occurrence of Antimicrobial Resistance in Fish-Pathogenic and Environmental Bacteria Associated with Four Danish Rainbow Trout Farms. *Appl. Environ. Microbiol.* **2000**, *66*, 4908–4915.
13. Zeng, S.S.; Hart, S.; Escobar, E.N.; Tesfai, K. Validation of Antibiotic Residue Tests for Dairy Goats. *J. Food Prot.* **1998**, *61*, 344–349.
14. Contreras, A.; Paape, M.J.; Di Carlo, A.L.; Miller, R.H.; Rainard, P. Evaluation of Selected Antibiotic Residue Screening Tests for Milk from Individual Goats. *J. Dairy Sci.* **1997**, *80*, 1113–1118.
15. Agmas, B.; Adugna, M. Antimicrobial Residue Occurrence and Its Public Health Risk of Beef Meat in Debre Tabor and Bahir Dar, Northwest Ethiopia. *Vet. World* **2018**, *11*, 902.
16. Chen, Y.; Zhang, H.; Luo, Y.; Song, J. Occurrence and Assessment of Veterinary Antibiotics in Swine Manures: A Case Study in East China. *Chin. Sci. Bull.* **2012**, *57*, 606–614.
17. Grote, M.; Schwake-Anduschus, C.; Michel, R.; Stevens, H.; Heyser, W.; Langenkamper, G.; Betsche, T.; Freitag, M. Incorporation of Veterinary Antibiotics into Crops from Manured Soil. *Landbauforsch. Volkenrode* **2007**, *57*, 25.
18. Moreno-Ortiz, V.C.; Martínez-Núñez, J.M.; Kravzov-Jinich, J.; Pérez-Hernández, L.A.; Moreno-Bonett, C.; Altagracia-Martínez, M. Los Medicamentos de Receta de Origen Sintético y Su Impacto En El Medio Ambiente. *Rev. Mex. Cienc. Farm.* **2013**, *44*, 17–29.
19. Ortiz, Z.; Vera, A.; Cayro Ch, J. Frequency of Beta-Lactams and Tetracyclines in Raw Milk in the Arequipa Milkshed. *Rev. Investig. Vet. Perú* **2008**, *19*, 140–143.
20. Vukovic, V.J.; Vicentijevic, M. Excretion of Chlortetracycline in Cow Milk Subsequent to Intrauterine Administration. *Philipp. J. Vet. Med.* **2016**, *53*, 135–138.
21. Darko, G.; Mensah, J.K.; Dapaah, S.S.; Odei, J. Estimated Dietary Exposure to Veterinary Residues in Chicken and Eggs. *Int. J. Food Contam.* **2015**, *2*, 1–8.
22. Kodimalar, K.; Rajini, R.A.; Ezhilvalavan, S.; Sarathchandra, G. A Survey of Chlortetracycline Concentration in Feed and Its Residue in Chicken Egg in Commercial Layer Farms. *J. Biosci.* **2014**, *39*, 425–431.
23. Ziani, K.; Pérez-López, M.; Mansouri, A.; Khaled, M.B.; Rodriguez, A.S.; Slimani, M. Assessment of Oxytetracycline Residue in Cooked and Raw Meat of Chicken Broilers before and after the End of Official Withdrawal Period. *Food Anal. Methods* **2018**, *11*, 2528–2537.
24. Billah, M.M.; Rana, S.M.; Hossain, M.S.; Ahamed, S.K.; Banik, S.; Hasan, M. Ciprofloxacin Residue and Their Impact on Biomolecules in Eggs of Laying Hens Following Oral Administration. *Int. J. Food Contam.* **2015**, *2*, 1–7.
25. Acevedo, D.; Montero, P.M.; Jaimes, J.D. Determinación de Antibióticos y Calidad Microbiológica de La Carne de Pollo Comercializada En Cartagena (Colombia). *Inf. Tecnológica* **2015**, *26*, 71–76.
26. Calderón, L.G.R.; Delgado, P.A.M.; Urbano, M.F.C.; Coy, F.A.C. Resistencia de La Salmonela a Los Antimicrobianos Convencionales Para Su Tratamiento. *Rev. CES Med. Vet. Zoote.* **2012**, *7*, 116–129.
27. Fortt, Z.A.; Cabello, C.F.; Buschmann, R.A. Residues of Tetracycline and Quinolones in Wild Fish Living around a Salmon Aquaculture Center in Chile. *Rev. Chil. DeInfectol. Organo Of. De La Soc. Chil. De Infectol.* **2007**, *24*, 14–18.
28. Yang, Y.; Cao, X.; Lin, H.; Wang, J. Antibiotics and Antibiotic Resistance Genes in Sediment of Honghu Lake and East Dongting Lake, China. *Microb. Ecol.* **2016**, *72*, 791–801.

29. Okocha, R.C.; Olatoye, I.O.; Adedeji, O.B. Food safety impacts of antimicrobial use and their residues in aquaculture. *Public Health Rev*. **2018**, *39*, 21. https://doi.org/10.1186/s40985-018-0099-2.
30. Lopez Cuevas, O.; Leon Felix, J.; Jimenez Edeza, M.; Chaidez Quiroz, C. Detection and Antibiotic Resistance of Escherichia Coli and Salmonella in Water and Agricultural Soil. *Rev. Fitotec. Mex.* **2009**, *32*, 119–126.
31. Montes, N.; Hijosa, M.; Bécares, E.; Méndez, R.; Martín-Villacorta, M. Presencia de Tetraciclinas En Purines y Estudio de Su Eliminación En Procesos de Fangos Activados. In *I Simp. Iberoam. Ing. Residuos. León, España*. **2008**. Available online: http://www.redisa.net/doc/artSim2008/agua/A10.pdf (accessed on 5 May 2020)
32. Hussein, A.E.R.Y.; Elmansoury, Y.H.; Hussien, M.O.; Taha, M.I.; Mahgoub, H.A.; El Hussein, A.M. Oxytetracycline Residues in Sheep Meat in Khartoum State, Sudan. *J. Adv. Vet. Anim. Res.* **2015**, *2*, 321–325.
33. Medina, M.S.; González, D.G.; Ramírez, A. Detección de Residuos Antimicrobianos En Tejidos Comestibles y Tetraciclina En Hueso de Cerdo. *Rev. Salud Anim.* **2008**, *30*, 110–115.
34. Hussein, M.A.; Khalil, S. Screening of Some Antibiotics and Anabolic Steroids Residues in Broiler Fillet Marketed in El-Sharkia Governorate. *Life Sci. J.* **2013**, *10*, 2111–2118.
35. Mohameda, H.; Anders, D.; Uswege, M.; Robinson, H. Occurrence and Distribution of Sulfonamides, Tetracyclines and Quinolones in Livestock Manure in Morogoro Municipality, Tanzania. *J. Zoonotic Dis. Public Health* **2017**, *22*, 4545–4554.
36. Darwish, W.S.; Eldaly, E.A.; El-Abbasy, M.T.; Ikenaka, Y.; Nakayama, S.; Ishizuka, M. Antibiotic Residues in Food: The African Scenario. *Japanese, J. Vet. Res.* **2013**, *61*, S13–S22.
37. Faleye, A.C.; Adegoke, A.A.; Ramluckan, K.; Bux, F.; Stenström, T.A. Antibiotic Residue in the Aquatic Environment: Status in Africa. *Open Chem. J.* **2018**, *16*, 890–903.
38. Restrepo Betancur, G.; Rodríguez Mazo, L.; Duque Cortés, J.E. Proliferación Microbiana y Calidad Posdescongelación de Semen Equino Criopreservado En Presencia de Antibióticos. *Rev. Investig. Vet. Perú* **2016**, *27*, 316–325.
39. Ujueta Rodríguez, S.; Araque Marín, A. Detection of Antimicrobial Residues in Muscle, Liver and Kidney of Pork for Sale in Bogota. *Rev. UDCA Actualidad Divulgación Científica.* **2016**, *19*, 371–379.
40. Ramirez, G.; Vélez, G.; Rondón, S. Determination of Antibiotic Residues and Withdrawal Time in Milk from the Town of Cartago, Valle Del Cauca. *Rev. Colomb. Cienc. Anim.* **2001**, *5*, 25–31.
41. Carrizales, K.B.M.; Gallardo, C.P.; Pérez, M.N.; Aguilar, D.G.G.; Arias, O.A.B. Resultados Parciales de La Determinación de Residuos de Antimicrobianos En Músculo y Riñón de Cerdos Sacrificados En Dos Rastros Municipales de La Zona Metropolitana de Guadalajara. *e-CUCBA* **2017**, *8*, 19–24.
42. Ortiz, Z.C.; Concha, U.A.; Cayro, C.J. Somatic Cell Counts in Raw Milk Contaminated with Antibiotic Residues. *Rev. Investig. Vet. Perú (RIVEP)* **2011**, *22*, 151–154.
43. del Carmen Briceño-Fereira, E.; Brito-Echenique, R.G.; Díaz-Rivera, H.Y.; Colina-Martinez, J.M.; Maniglia-Mérida, G.C.; Arrieta-Mendoza, D. Determinación de Residuos de Cloranfenicol y Sulfamidas En Leches de Larga Duración, En La Ciudad de Maracay, Venezuela. *Rev. Cient.* **2018**, *2*, 121–128.
44. Xie, H.; Wang, X.; Chen, J.; Li, X.; Jia, G.; Zou, Y.; Zhang, Y.; Cui, Y. Occurrence, Distribution and Ecological Risks of Antibiotics and Pesticides in Coastal Waters around Liaodong Peninsula, China. *Sci. Total Environ.* **2019**, *656*, 946–951.
45. Karthikeyan, K.G.; Meyer, M.T. Occurrence of Antibiotics in Wastewater Treatment Facilities in Wisconsin, USA. *Sci. Total Environ.* **2006**, *361*, 196–207.
46. Xu, W.; Yan, W.; Li, X.; Zou, Y.; Chen, X.; Huang, W.; Miao, L.; Zhang, R.; Zhang, G.; Zou, S. Antibiotics in Riverine Runoff of the Pearl River Delta and Pearl River Estuary, China: Concentrations, Mass Loading and Ecological Risks. *Environ. Poll.* **2013**, *182*, 402–407.
47. Santiago, M.L.; Espinosa, A.; del Carmen Bermúdez, M. Uso de Antibióticos En La Camaronicultura. *Rev. Mex. Cienc. Farm.* **2009**, *40*, 22–32.
48. Ramos, A. Physic-Chemical Properties of Human Consumption Drugs in Water. *Rev. Cuba. Hig. Epidemiología* **2009**, *47(2)*. Available online: http://scielo.sld.cu/scielo.php?script=sci_arttext&pid=S1561-30032009000200008&lng=es&nrm=iso. ISSN 1561-3003. (accessed on 7 May 2020)
49. Tejada, C.; Quiñonez, E.; Peña, M. Contaminantes Emergentes En Aguas: Metabolitos de Fármacos. Una Revisión. *Rev. Facultad Cienc. Básicas* **2014**, *10*, 80–101.
50. Do, M.H.N.; Yamaguchi, T.; Okihashi, M.; Harada, K.; Konishi, Y.; Uchida, K.; Bui, L.T.; Nguyen, T.D.; Phan, H.B.; Bui, H.D.T. Screening of Antibiotic Residues in Pork Meat in Ho Chi Minh City, Vietnam, Using a Microbiological Test Kit and Liquid Chromatography/Tandem Mass Spectrometry. *Food Control* **2016**, *69*, 262–266.
51. Carrillo, H.H.G.; Plascencia, A.E.; Barbeitia, G.C.; del Almada, M.C.B. Estabilidad de Sulfametazina En Carne y Productos Cárnicos de Cerdo Tratados Térmicamente. *Vet. Méx.* **2004**, *35*, 91–100.
52. Escobedo, Y.; Espinosa, A.; del Refugio Robles, M.; del Carmen Bermúdez, M. Transformación y Acumulación de Sulfametazina En Porcinos Alimentados Con Una Dieta Medicada. *Rev. Mex. Cienc. Farm.* **2007**, *38*, 5–13.
53. Yang, Y.; Owino, A.A.; Gao, Y.; Yan, X.; Xu, C.; Wang, J. Occurrence, Composition and Risk Assessment of Antibiotics in Soils from Kenya, Africa. *Ecotoxicology* **2016**, *25*, 1194–1201.
54. Nirala, R.; Anjana, K.; Mandal, K.; Jayachandran, C. Antibiotic Residue-Food Producing Animal Origin and Its Impact on Human Health. *Int. J. Livest. Res.* **2018**, *8*, 61–69.
55. Jayalakshmi, K.; Paramasivam, M.; Sasikala, M.; Tamilam, T.V.; Sumithra, A. Review on Antibiotic Residues in Animal Products and Its Impact on Environments and Human Health. *J. Entomol. Zool. Stud.* **2017**, *5*, 1446–1451.
56. Tamtam, F.; Mercier, F.; Le Bot, B.; Eurin, J.; Dinh, Q.T.; Clément, M.; Chevreuil, M. Occurrence and Fate of Antibiotics in the Seine River in Various Hydrological Conditions. *Sci. Total Environ.* **2008**, *393*, 84–95.

57. Mutiyar, P.K.; Mittal, A.K. Risk Assessment of Antibiotic Residues in Different Water Matrices in India: Key Issues and Challenges. *Environ. Sci. Pollut. Res.* **2014**, *21*, 7723–7736.
58. Li, X.; Shi, H.; Li, K.; Zhang, L.; Gan, Y. Occurrence and Fate of Antibiotics in Advanced Wastewater Treatment Facilities and Receiving Rivers in Beijing, China. *Front. Environ. Sci. Eng.* **2014**, *8*, 888–894.
59. Agoba, E.E.; Adu, F.; Agyare, C.; Boamah, V.E. Antibiotic Use and Practices in Selected Fish Farms in the Ashanti Region of Ghana. *J. Infect. Dis. Treatment* **2017**, *3*, 2472–1093.
60. Alvarado, S.M.; Ascanio, E.; Méndez, C. Determinación de Residuos de Oxitetraciclina En Muestras de Tejido Bovino Destinadas al Consumo Humano. *Rev. Fac Cienc. Vet.* **2008**, *49*, 73–79.
61. Tajik, H; Malekinejad, H; Razavi-Rouhani, S.M; Pajouhi, M.R; Mahmoudi, R; Haghnazari A. Chloramphenicol residues in chicken liver, kidney and muscle: a comparison among the antibacterial residues monitoring methods of Four Plate Test, ELISA and HPLC. *Food Chem Toxicol*. **2010,** 48(8-9), 2464-8. doi: 10.1016/j.fct.2010.06.014.
62. Bele, C.; Matea, C.T.; Dulf, F.; Miclean, M. Determination of Six Sulfonamides in Pork and Beef Meat by a New Solid Phase Extraction and HPLC-UV for Detection. *Bull. USAMV-CN* **2007**, *64*, 1–2.
63. Del Bermúdez Almada, M.C.; Miranda de Contreras, L.; Espinosa Plascencia, A.; Valenzuela Quintanar, A.I.; Vázquez Moreno, L. *Residuos de Sulfonamidas En Músculo de Porcinos Sacrificados En La Región Noroeste de México*;Rev. Cient. Fac. Cien .Vet-LUZ. **2001**.11,127−132. Available online: https://produccioncientificaluz.org/index.php/cientifica/article/view/14760/14737
64. Jia, A.; Wan, Y.; Xiao, Y.; Hu, J. Occurrence and Fate of Quinolone and Fluoroquinolone Antibiotics in a Municipal Sewage Treatment Plant. *Water Res.* **2012**, *46*, 387–394.
65. Batt, A.L.; Snow, D.D.; Aga, D.S. Occurrence of Sulfonamide Antimicrobials in Private Water Wells in Washington County, Idaho, USA. *Chemosphere* **2006**, *64*, 1963–1971.
66. Abebew, D.; Belihu, K.; Zewde, G. Detection and Determination of Oxytetracycline and Penicillin G Antibiotic Residue Levels in Bovine Bulk Milk from Nazareth Dairy Farms, Ethiopia. *Ethiop. Vet. J.* **2014**, *18*, 1–15.
67. Tao, X.; Jiang, H.; Zhu, J.; Niu, L.; Wu, X.; Shi, W.; Wang, Z.; Shen, J. Detection of Ultratrace Chloramphenicol Residues in Milk and Chicken Muscle Samples Using a Chemiluminescent ELISA. *Anal. Lett.* **2012**, *45*, 1254–1263.
68. Kang, J.; Park, S.J.; Park, H.C.; Hossain, M.A.; Kim, M.A.; Son, S.W.; Lim, C.M.; Kim, T.W.; Cho, B.H. Multiresidue screening of veterinary drugs in meat, milk, egg, and fish using liquid chromatography coupled with ion trap time-of-flight mass spectrometry. *Biotechnol Appl Biochem*. **2016**, *182*, 635–652. doi: 10.1007/s12010-016-2350-y.
69. Sultan, I.A. Detection of Enrofloxacin Residue in Livers of Livestock Animals Obtained from a Slaughterhouse in Mosul City. *J. Vet. Sci. Technol.* **2014**, *5*,168. doi:10.4172/2157-7579.1000168
70. Zielezny, Y.; Groeneweg, J.; Vereecken, H.; Tappe, W. Impact of Sulfadiazine and Chlorotetracycline on Soil Bacterial Community Structure and Respiratory Activity. *Soil Biol. Biochem.* **2006**, *38*, 2372–2380.
71. Hammesfahr, U.; Heuer, H.; Manzke, B.; Smalla, K.; Thiele-Bruhn, S. Impact of the Antibiotic Sulfadiazine and Pig Manure on the Microbial Community Structure in Agricultural Soils. *Soil Biol. Biochem.* **2008**, *40*, 1583–1591.
72. Carballo, M.; Aguayo, S.; González, M.; Esperon, F.; de la Torre, A. Environmental Assessment of Tetracycline's Residues Detected in Pig Slurry and Poultry Manure. *J. Environ. Prot.* **2016**, *7*, 82–92.
73. Abbasi, M.M.; Nemati, M.; Babaei, H.; Ansarin, M.; Nourdadgar, A.-O.-S. Solid-Phase Extraction and Simultaneous Determination of Tetracycline Residues in Edible Cattle Tissues Using an HPLC-FL Method. *Iranian, J. Pharm. Res. IJPR* **2012**, *11*, 781.
74. Gaurav, A.; Gill, J.P.S.; Aulakh, R.S.; Bedi, J.S. ELISA based monitoring and analysis of tetracycline residues in cattle milk in various districts of Punjab. *Vet. World* **2014**, *7*, 26–29.
75. Plozza, T.; Trenerry, V.C.; Zeglinski, P.; Nguyen, H.; Johnstone, P. The Confirmation and Quantification of Selected Aminoglycoside Residues in Animal Tissue and Bovine Milk by Liquid Chromatography Tandem Mass Spectrometry. *Int. Food Res. J.* **2011**, 18, 1077−1084.
76. Şenyuva, H.Z.; Özden, T.; Sarica, D.Y. High-Performance Liquid Chromatographic Determination of Oxytetracycline Residue in Cured Meat Products. *Turk. J. Chem.* **2000**, *24*, 395–400.
77. Hou, J.; Wan, W.; Mao, D.; Wang, C.; Mu, Q.; Qin, S.; Luo, Y. Occurrence and Distribution of Sulfonamides, Tetracyclines, Quinolones, Macrolides, and Nitrofurans in Livestock Manure and Amended Soils of Northern China. *Environ. Sci. Pollut. Res.* **2015**, *22*, 4545–4554.
78. Jacobsen, A.M.; Halling-Sørensen, B.; Ingerslev, F.; Hansen, S.H. Simultaneous Extraction of Tetracycline, Macrolide and Sulfonamide Antibiotics from Agricultural Soils Using Pressurised Liquid Extraction, Followed by Solid-Phase Extraction and Liquid Chromatography–Tandem Mass Spectrometry. *J. Chromatogr. A* **2004**, *1038*, 157–170.
79. Karcı, A.; Balcıoğlu, I.A. Investigation of the Tetracycline, Sulfonamide, and Fluoroquinolone Antimicrobial Compounds in Animal Manure and Agricultural Soils in Turkey. *Sci. Total Environ.* **2009**, *407*, 4652–4664.
80. Li, Y.-W.; Wu, X.-L.; Mo, C.-H.; Tai, Y.-P.; Huang, X.-P.; Xiang, L. Investigation of Sulfonamide, Tetracycline, and Quinolone Antibiotics in Vegetable Farmland Soil in the Pearl River Delta Area, Southern China. *J. Agr. Food Chem.* **2011**, *59*, 7268–7276.
81. Liu, B.; Li, Y.; Zhang, X.; Wang, J.; Gao, M. Effects of Chlortetracycline on Soil Microbial Communities: Comparisons of Enzyme Activities to the Functional Diversity via Biolog EcoPlates™. *Eur. J. Soil Biol.* **2015**, *68*, 69–76.
82. Thiele-Bruhn, S.; Beck, I.-C. Effects of Sulfonamide and Tetracycline Antibiotics on Soil Microbial Activity and Microbial Biomass. *Chemosphere* **2005**, *59*, 457–465.

83. Wu, X.-L.; Xiang, L.; Yan, Q.-Y.; Jiang, Y.-N.; Li, Y.-W.; Huang, X.-P.; Li, H.; Cai, Q.-Y.; Mo, C.-H. Distribution and Risk Assessment of Quinolone Antibiotics in the Soils from Organic Vegetable Farms of a Subtropical City, Southern China. *Sci. Total Environ.* **2014**, *487*, 399–406.
84. Zhang, Z.; Sun, K.; Gao, B.; Zhang, G.; Liu, X.; Zhao, Y. Adsorption of Tetracycline on Soil and Sediment: Effects of PH and the Presence of Cu (II). *J. Hazard. Mater.* **2011**, *190*, 856–862.
85. Shao, B.; Dong, D.; Wu, Y.; Hu, J.; Meng, J.; Tu, X.; Xu, S. Simultaneous Determination of 17 Sulfonamide Residues in Porcine Meat, Kidney and Liver by Solid-Phase Extraction and Liquid Chromatography–Tandem Mass Spectrometry. *Anal. Chim. Acta* **2005**, *546*, 174–181.
86. Hela, W.; Brandtner, M.; Widek, R.; Schuh, R. Determination of Sulfonamides in Animal Tissues Using Cation Exchange Reversed Phase Sorbent for Sample Cleanup and HPLC–DAD for Detection. *Food Chem.* **2003**, *83*, 601–608.
87. Biehl, L.G.; Bevill, R.F.; Limpoka, M.; Koritz, G.D. Sulfamethazine Residues in Swine. *J. Vet. Pharmacol. Ther.* **1981**, *4*, 285–290.
88. Pecorelli, I.; Bibi, R.; Fioroni, L.; Galarini, R. Validation of a Confirmatory Method for the Determination of Sulphonamides in Muscle According to the European Union Regulation 2002/657/EC. *J. Chromatogr. A* **2004**, *1032*, 23–29.
89. Whipple, D.M.; Samuelson, G.; Heath, G.E.; Showalter, D.H. Tissue Residue Depletion and Recycling of Sulfamethazine in Swine. *J. Am. Vet. Med. Assoc.* **1980**, *176*, 1348–1352.
90. Saschenbrecker, P.W.; Fish, N.A. Sulfamethazine Residues in Uncooked Edible Tissues of Port Following Recommended Oral Administration and Withdrawal. *Can. J. Comp. Med.* **1980**, *44*, 338.
91. Ashworth, R.B.; Epstein, R.L.; Thomas, M.H.; Frobish, L.T. Sulfamethazine Blood/Tissue Correlation Study in Swine. *Am. J. Vet. Res.* **1986**, *47*, 2596–2603.
92. Smallidge, R.L.; Kentzer, E.J.; Stringham, K.R.; Kim, E.H.; Lehe, C.; Stringham, R.W.; Mundell, E.C. Sulfamethazine and Sulfathiazole Determination at Residue Levels in Swine Feeds by Reverse-Phase Liquid Chromatography with Post-Column Derivatization. *J. Assoc. Off. Anal. Che.* **1988**, *71*, 710–717.
93. Elizabeta, D.S.; Zehra, H.M.; Biljana, S.D.; Pavle, S.; Risto, U. Screening of veterinary drug residues in milk from individual farms in Macedonia. *Mac Vet Rev.* **2011**, 34:5–13.
94. Rajaian, H.; Symonds, H.W.; Bowmer, C.J. Pharmacokinetics of Sulphamethazine and Its Acetyl Metabolite in Chickens. *J. Appl. Anim. Re.* **2001**, *19*, 85–96.
95. Long, A.R.; Hsieh, L.C.; Malbrough, M.S.; Short, C.R.; Barker, S.A. Multiresidue Method for the Determination of Sulfonamides in Pork Tissue. *J. Agr. food Chem.* **1990**, *38*, 423–426.
96. Epstein, R.L.; Randecker, V.; Corrao, P.; Keeton, J.T.; Cross, H.R. Influence of Heat and Cure Preservatives on Residues of Sulfamethazine, Chloramphenicol, and Cyromazine in Muscle Tissue. *J. Agr. Food Chem.* **1988**, *36*, 1009–1012.
97. Sánchez, R.L.; Fuentes, H.V.O.; Sumano, L.H.; Reza, G.C. Detección de Residuos de Sulfonamidas En Carne y Vísceras de Bovinos Sacrificados. *Cebú* **1988**, *14*, 72–78.
98. Chee-Sanford, J.C.; Aminov, R.I.; Krapac, I.J.; Garrigues-Jeanjean, N.; Mackie, R.I. Occurrence and Diversity of Tetracycline Resistance Genes in Lagoons and Groundwater Underlying Two Swine Production Facilities. *Appl. Environ. Microbiol.* **2001**, *67*, 1494–1502.
99. Coyne, R.; Hiney, M.; O'Connor, B.; Kerry, J.; Cazabon, D.; Smith, P. Concentration and Persistence of Oxytetracycline in Sediments under a Marine Salmon Farm. *Aquaculture* **1994**, *123*, 31–42.
100. Golet, E.M.; Xifra, I.; Siegrist, H.; Alder, A.C.; Giger, W. Environmental Exposure Assessment of Fluoroquinolone Antibacterial Agents from Sewage to Soil. *Environ. Sci. Technol.* **2003**, *37*, 3243–3249.
101. Lindsey, M.E.; Meyer, M.; Thurman, E.M. Analysis of Trace Levels of Sulfonamide and Tetracycline Antimicrobials in Groundwater and Surface Water Using Solid-Phase Extraction and Liquid Chromatography/Mass Spectrometry. *Anal. Chem.* **2001**, *73*, 4640–4646.
102. McArdell, C.S.; Molnar, E.; Suter, M.J.-F.; Giger, W. Occurrence and Fate of Macrolide Antibiotics in Wastewater Treatment Plants and in the Glatt Valley Watershed, Switzerland. *Environ. Sci. Technol.* **2003**, *37*, 5479–5486.
103. Ascanio, E.; Sogbe, E.; Meléndez, B.; Briceño, E.; Diaz, C.; Ascanio, D.; Ascanio, D. Chloramphenicol Residues in Comercial Milk Produced for Human Consumption in Venezuela. *J. Vet. Pharmacol. Ther.* **2006**, *29* (Suppl. 1), 173–174.
104. Allara, M.; Izquierdo, P.; Torres Ferrari, G; Rodríguez, B. Penicilina G En Leche Pasteurizada Producida En El Estado Zulia-Venezuela. Cient. Fac. Cien .Vet-LUZ. **2002**, *12*,683–687. Available online: http://www.saber.ula.ve/handle/123456789/27727
105. Gomez, M.; de Quintana, G.A.; De Nobrega, J.; Alvarado, S. Determinación de Residuos de Tetraciclinas (Oxitetraciclina, Tetraciclina, Demeclociclina, Clortetraciclina y Doxiciclina) En Leches Líquidas Pasteurizadas, En Polvo y En Fórmulas Infantiles de Venta En Mercados Del Área Metropolitana. *Rev. Fac. Farmacia.* **2016**, *79*, 77–86.
106. Guerrero, D.M.; Motta, R.; Gamarra, G.; Benavides, E.R.; Roque, M.; Salazar, M.E. Detección de Residuos de Antibióticos β-Lactámicos y Tetraciclinas En Leche Cruda Comercializada En El Callao. *Cienc. Invest.* **2009**, *12*, 79–82.
107. Hamscher, G.; Sczesny, S.; Höper, H.; Nau, H. Determination of Persistent Tetracycline Residues in Soil Fertilized with Liquid Manure by High-Performance Liquid Chromatography with Electrospray Ionization Tandem Mass Spectrometry. *Anal. Chem.* **2002**, *74*, 1509–1518.
108. Abd El Monem, M.K.; Soliman, M.R.; Saad, S.M. Oxytetracycline Residues in Broiler Carcasses Produced by Closed and Open System. *J. Egypt. Vet. Med. Assoc.***2002**, *62*, 119–124.
109. Gigosos, P.G.; Revesado, P.R.; Cadahıa, O.; Fente, C.A.; Vazquez, B.I.; Franco, C.M.; Cepeda, A. Determination of Quinolones in Animal Tissues and Eggs by High-Performance Liquid Chromatography with Photodiode-Array Detection. *J. Chromatogr. A.* **2000**, *871*, 31–36.

110. Javadi, A.; Mirzaie, H.; Khatibi, S. Effect of Roasting, Boiling and Microwaving Cooking Methods on Enrofloxacin Residues in Edible Tissues of Broiler. *Afr. J. Pharm. Pharmacol.* **2011**, *5*, 214–218.
111. Lolo, M; Pedreira, S; Vázquez Belda, B; Miranda J.M; Franco, C; Cepeda, A; Asuncion Fente C. The Effect of Cooking on Enrofloxacin Residues in Chicken Muscle. *Food Addit. Contam*. **2006**.23, 988–993. doi 10.1080/02652030600904894
112. Salehzadeh, F.; Salehzadeh, A.; Rokni, N.; Madani, R.; Golchinefar, F. Enrofloxacin Residue in Chicken Tissues from Tehran Slaughterhouses in Iran. *Pak. J. Nutr.* **2007**, *6*, 409–413.
113. Salehzadeh, F.; Madani, R.; Salehzadeh, A.; Rokni, N.; Golchinefar, F. Oxytetracycline Residue in Chicken Tissues from Tehran Slaughterhouses in Iran. *Pak. J. Nutr.* **2006**, *5*, 377–381.
114. Al-Mustafa, Z.H.; Al-Ghamdi, M.S. Use of Norfloxacin in Poultry Production in the Eastern Province of Saudi Arabia and Its Possible Impact on Public Health. *Int. J Environ. Health Res.* **2000**, *10*, 291–299.
115. Myllyniemi, A.-L.; Rannikko, R.; Lindfors, E.; Niemi, A.; Bäckman, C. Microbiological and Chemical Detection of Incurred Penicillin G, Oxytetracycline, Enrofloxacin and Ciprofloxacin Residues in Bovine and Porcine Tissues. *Food Addit. Contam.* **2000**, *17*, 991–1000.
116. Kirbiš, A. Microbiological Screening Method for Detection of Aminoglycosides, β-Lactames, Macrolides, Tetracyclines and Quinolones in Meat Samples. *Slov. Vet. Res.* **2007**, *44*, 11–18.
117. Errecalde, J.O. Uso de Antimicrobianos En Animales de Consumo: Incidencia Del Desarrollo de Resistencias En Salud Pública; FAO: Roma, Italy, 2004.
118. Guest, G. Food Animal Drug Residues. *Vet. Med.* **1998**, *83*, 404–416.
119. Kim, M.; Cho, B.H.; Lim, C.M.; Kim, D.G.; Yune, S.Y.; Shin, J.Y.; Bong, Y.H.; Kang, J.; Kim, M.A.; Son S.W. Chemical residues and contaminants in foods of animal origin in Korea during the past decade. *J Agric Food Chem*. **2013**, 61(10),2293-8.
120. Koenen-Dierick, K.; Okerman, L.; De Zutter, L.; Degroodt, J.M.; Van Hoof, J.; Srebrnik, S. A One-Plate Microbiological Screening Test for Antibiotic Residue Testing in Kidney Tissue and Meat: An Alternative to the EEC Four-Plate Method? *Food Addit. Contam.* **1995**, *12*, 77–82.
121. Korner, U.; Kuhne, M.; Wenzel, S. Tetracycline Residues in Meat and Bone Meals. Part 1: Methodology and Examination of Field Samples. *Food Addit. Contam.* **2001**, *18*, 293–302.
122. Korsrud, G.O.; Salisbury, C.D.; Fesser, A.C.; MacNeil, J.D. Laboratory Evaluation of the Charm Farm Test for Antimicrobial Residues in Meat. *J. Food Prot.* **1995**, *58*, 1129–1132.
123. Kühne, M.; Wegmann, S.; Kobe, A.; Fries, R. Tetracycline Residues in Bones of Slaughtered Animals. *Food Control.* **2000**, *11*, 175–180.
124. Kühne, M.; Mitzscherling, A.T. Zum Eintrag von Gebundenen Tetracyclin-Rückständen in Die Nahrungskette-Ein Beitrag Zur Gefahrenidentifikation.Berl. Münch. Tierätzl. In *Bound Residues of Tetracyclines in the Food Chain-a Contribution to the Hazard Identification*. Schlütersche Verlagsgesellschaft mbH & Co. KG.**2004,** *117*, 2–7**.** Available online: https://www.vetline.de/zum-eintrag-von-gebundenen-tetracyclin-rueckstaenden-in-die-nahrungskette-ein-beitrag-zur (accessed on 18 May 2020)
125. Macauley, J.J.; Qiang, Z.; Adams, C.D.; Surampalli, R.; Mormile, M.R. Disinfection of Swine Wastewater Using Chlorine, Ultraviolet Light and Ozone. *Water Res.* **2006**, *40*, 2017–2026.
126. Organization, W.H. Impacts of Antimicrobial Growth Promoter Termination in Denmark. In Proceedings of the The WHO International Review Panel's Evaluation of the Termination of the Use of Antimicrobial Growth Promoters in Denmark, Foulum, Denmark, 6–9 November 2002.
127. Reemtsma, T.; Jekel, M. Organic Pollutants in the Water Cycle: Properties, Occurrence, Analysis and Environmental Relevance of Polar Compounds.Eds, Wiley-VCH. 1st edition. **2006,** ISBN-13: 978-3527312979 doi:10.1002/352760877X
128. Ingerslev, F.; Halling-Sørensen, B. Biodegradability of Metronidazole, Olaquindox, and Tylosin and Formation of Tylosin Degradation Products in Aerobic Soil–Manure Slurries. *Ecotox. Environ. Saf.* **2001**, *48*, 311–320.
129. Loke, M.-L.; Tjørnelund, J.; Halling-Sørensen, B. Determination of the Distribution Coefficient (LogKd) of Oxytetracycline, Tylosin A, Olaquindox and Metronidazole in Manure. *Chemosphere* **2002**, *48*, 351–361.
130. Malintan, N.T.; Mohd, M.A. Determination of Sulfonamides in Selected Malaysian Swine Wastewater by High-Performance Liquid Chromatography. *J. Chromatogr. A* **2006**, *1127*, 154–160.
131. Berrada, H.; Borrull, F.; Font, G.; Moltó, J.C.; Marcé, R.M. Validation of a Confirmatory Method for the Determination of Macrolides in Liver and Kidney Animal Tissues in Accordance with the European Union Regulation 2002/657/EC. *J. Chromatogr. A* **2007**, *1157*, 281–288.
132. Loke, M.-L.; Jespersen, S.; Vreeken, R.; Halling-Sørensen, B.; Tjørnelund, J. Determination of Oxytetracycline and Its Degradation Products by High-Performance Liquid Chromatography–Tandem Mass Spectrometry in Manure-Containing Anaerobic Test Systems. *J. Chromatogr. B* **2003**, *783*, 11–23.
133. Höper, H.; Kues, J.; Nau, H.; Hamscher, G. Eintrag Und Verbleib von Tierarzneimittelwirkstoffen in Böden. *Bodenschutz* **2002**, *4*, 141–148.
134. Aga, D.S.; Goldfish, R.; Kulshrestha, P. Application of ELISA in Determining the Fate of Tetracyclines in Land-Applied Livestock Wastes. *Analyst* **2003**, *128*, 658–662.
135. De Liguoro, M.; Cibin, V.; Capolongo, F.; Halling-Sørensen, B.; Montesissa, C. Use of Oxytetracycline and Tylosin in Intensive Calf Farming: Evaluation of Transfer to Manure and Soil. *Chemosphere* **2003**, *52*, 203–212.
136. Baquero, F.; Martínez, J.-L.; Cantón, R. Antibiotics and Antibiotic Resistance in Water Environments. *Curr. Opin Biotechnol.* **2008**, *19*, 260–265.

137. Kumar, K.; Gupta, S.C.; Chander, Y.; Singh, A.K. Antibiotic Use in Agriculture and Its Impact on the Terrestrial Environment. *Adv. Agron.* **2005**, *87*, 1–54.
138. Chen, K.; Zhou, J.L. Occurrence and Behavior of Antibiotics in Water and Sediments from the Huangpu River, Shanghai, China. *Chemosphere* **2014**, *95*, 604–612.
139. Khan, G.A.; Berglund, B.; Khan, K.M.; Lindgren, P.-E.; Fick, J. Occurrence and Abundance of Antibiotics and Resistance Genes in Rivers, Canal and near Drug Formulation Facilities–a Study in Pakistan. *PLoS ONE* **2013**, *8*, e62712.
140. Luo, Y.; Xu, L.; Rysz, M.; Wang, Y.; Zhang, H.; Alvarez, P.J. Occurrence and Transport of Tetracycline, Sulfonamide, Quinolone, and Macrolide Antibiotics in the Haihe River Basin, China. *Environ. Sci. Technol.* **2011**, *45*, 1827–1833.
141. Li, W.; Shi, Y.; Gao, L.; Liu, J.; Cai, Y. Occurrence of Antibiotics in Water, Sediments, Aquatic Plants, and Animals from Baiyangdian Lake in North China. *Chemosphere* **2012**, *89*, 1307–1315.
142. Zhu, S.; Chen, H.; Li, J. Sources, Distribution and Potential Risks of Pharmaceuticals and Personal Care Products in Qingshan Lake Basin, Eastern China. *Ecotox. Environ. Saf.* **2013**, *96*, 154–159.
143. Zhou, L.-J.; Ying, G.-G.; Liu, S.; Zhao, J.-L.; Yang, B.; Chen, Z.-F.; Lai, H.-J. Occurrence and Fate of Eleven Classes of Antibiotics in Two Typical Wastewater Treatment Plants in South China. *Sci. Total Environ.* **2013**, *452*, 365–376.
144. Nagulapally, S.R.; Ahmad, A.; Henry, A.; Marchin, G.L.; Zurek, L.; Bhandari, A. Occurrence of Ciprofloxacin-, Trimethoprim-Sulfamethoxazole-, and Vancomycin-Resistant Bacteria in a Municipal Wastewater Treatment Plant. *Water Environ. Res.* **2009**, *81*, 82–90.
145. Michael, I.; Rizzo, L.; McArdell, C.S.; Manaia, C.M.; Merlin, C.; Schwartz, T.; Dagot, C.; Fatta-Kassinos, D. Urban Wastewater Treatment Plants as Hotspots for the Release of Antibiotics in the Environment: A Review. *Water Res.* **2013**, *47*, 957–995.
146. Soto, D.; Norambuena, F. Evaluation of Salmon Farming Effects on Marine Systems in the Inner Seas of Southern Chile: A Large-Scale Mensurative Experiment. *J. Appl. Ichthyol.* **2004**, *20*, 493–501.
147. Cabello, F.C. Antibiotics and Aquaculture in Chile: Implications for Human and Animal Health. *Rev. Med. Chil.* **2004**, *132*, 1001–1006.
148. Cabello, F.C. Antibiotics and Aquaculture in Chile: implications for human and animal health. *Rev.Med. Chil.* **2004**, *132*, 1001−6 doi: 10.4067/s0034-98872004000800014.
149. Björklund, H.; Bondestam, J.; Bylund, G. Residues of Oxytetracycline in Wild Fish and Sediments from Fish Farms. *Aquaculture* **1990**, *86*, 359–367.
150. Grave, K.; Lingaas, E.; Bangen, M.; Rønning, M. Surveillance of the Overall Consumption of Antibacterial Drugs in Humans, Domestic Animals and Farmed Fish in Norway in 1992 and 1996. *J. Antimicrob. Chemother.* **1999**, *43*, 243–252.
151. Buschmann, A.H.; Riquelme, V.A.; Hernández-González, M.C.; Varela, D.; Jiménez, J.E.; Henríquez, L.A.; Vergara, P.A.; Guíñez, R.; Filún, L. A Review of the Impacts of Salmonid Farming on Marine Coastal Ecosystems in the Southeast Pacific. *ICES J. Mar. Sci.* **2006**, *63*, 1338–1345.
152. Hektoen, H.; Berge, J.A.; Hormazabal, V.; Yndestad, M. Persistence of Antibacterial Agents in Marine Sediments. *Aquaculture* **1995**, *133*, 175–184.
153. Samuelsen, O.B.; Lunestad, B.T.; Husevåg, B.; Hølleland, T.; Ervik, A. Residues of Oxolinic Acid in Wild Fauna Following Medication in Fish Farms. *Dis. Aquat. Org.* **1992.** *12*, 111−119. doi.org/10.3354/dao012111
154. Sørum, H. Antimicrobial Drug Resistance in Fish Pathogens. *In Antimicrobial Resistance in Bacteria of Animal Origin*.Eds. Aarestrup, F.M. **2005**, 213–238. doi.org/10.1128/9781555817534.ch13
155. Coyne, R.; Hiney, M.; Smith, P. Transient Presence of Oxytetracycline in Blue Mussels (Mytilus Edulis) Following Its Therapeutic Use at a Marine Atlantic Salmon Farm. *Aquaculture* **1997**, *149*, 175–181.
156. Kerry, J.; Coyne, R.; Gilroy, D.; Hiney, M.; Smith, P. Spatial Distribution of Oxytetracycline and Elevated Frequencies of Oxytetracycline Resistance in Sediments beneath a Marine Salmon Farm Following Oxytetracycline Therapy. *Aquaculture* **1996**, *145*, 31–39.
157. Reveurs, T.; Díaz, R. *Método de Determinación de Tetraciclinas En Tejido Por HPLC-Diode-Array*; Centro Nacional de Alimentación. Instituto de Salud Carlos III: Madrid, España, 1994.
158. Degroodt, J.M.; de Bukanski, B.W.; Srebrnik, S. Oxolinic Acid and Flumequine in Fish Tissues: Validation of an HPLC Method; Analysis of Medicated Fish and Commercial Fish Samples. *J. Liq. Chromatogr. Relat. Technol.* **1994**, *17*, 1785–1794.
159. Codex Alimentarius. Alimentarius, C. Norma General Del Codex Para Los Contaminantes y Las Toxinas Presentes En Los Alimentos. *Codex Stan.* FAO/ OMS. **2019**. 30–76. Available online: https://www.fao.org/fao-who-codexalimentarius/sh-proxy/en/?lnk=1&url=https%253A%252F%252Fworkspace.fao.org%252Fsites%252Fcodex%252FStandards%252FCXS%2B193-1995%252FCXS_193s.pdf (accessed on 19 May 2020)
160. Han, R.W.; Zheng, N.; Yu, Z.N.; Wang, J.; Xu, X.M.; Qu, X.Y.; Li, S.L.; Zhang, Y.D.; Wang, J.Q. Simultaneous determination of 38 veterinary antibiotic residues in raw milk by UPLC-MS/MS. *Food Chem*. **2015**, 181, 119–126. doi: 10.1016/j.foodchem.2015.02.041.
161. De Grandis, S.A.; Stevenson, R.M. Antimicrobial Susceptibility Patterns and R Plasmid-Mediated Resistance of the Fish Pathogen Yersinia Ruckeri. *Antimicrob. Agents Chemother.* **1985**, *27*, 938–942.
162. DePaola, A.; Flynn, P.A.; McPhearson, R.M.; Levy, S. Phenotypic and Genotypic Characterization of Tetracycline-and Oxytetracycline-Resistant Aeromonas Hydrophila from Cultured Channel Catfish (Ictalurus Punctatus) and Their Environments. *Appl. Environ. Microbiol.* **1988**, *54*, 1861–1863.
163. Furones, M.D.; Gilpin, M.L.; Munn, C.B. Culture Media for the Differentiation of Isolates of Yersinia Ruckeri, Based on Detection of a Virulence Factor. *J. Appl. Bacteriol.* **1993**, *74*, 360–366.

164. Guardabassi, L.; Dalsgaard, A.; Raffatellu, M.; Olsen, J.E. Increase in the Prevalence of Oxolinic Acid Resistant Acinetobacter Spp. Observed in a Stream Receiving the Effluent from a Freshwater Trout Farm Following the Treatment with Oxolinic Acid-Medicated Feed. *Aquaculture* **2000**, *188*, 205–218.
165. Halling-Sørensen, B.; Nors Nielsen, S.; Lanzky, P.F.; Ingerslev, F.; Holten Lützhøft, H.C.; Jørgensen, S.E. Occurrence, Fate and Effects of Pharmaceutical Substances in the Environment—A Review. *Chemosphere* **1998**, *36*, 357–393. https://doi.org/10.1016/S0045-6535(97)00354-8.
